# Immune-Related Genes of *Megalurothrips usitatus* (Bagrall) Against *Beauveria brongniartii* and *Akanthomyces attenuatus* Identified Using RNA Sequencing

**DOI:** 10.3389/fphys.2021.671599

**Published:** 2021-08-11

**Authors:** Yueyin Chen, Bo Yang, Zhaoyang Li, Yang Yue, Qingheng Tian, Weiyi Chen, Shaukat Ali, Jianhui Wu

**Affiliations:** ^1^Key Laboratory of Bio-Pesticide Innovation and Application, Engineering Research Center of Biological Control, College of Plant Protection, South China Agricultural University, Guangzhou, China; ^2^Agricultural Genomics Institute at Shenzhen, Chinese Academy of Agricultural Sciences, Shenzhen, China; ^3^Key Laboratory of Integrated Pest Management on Tropical Crops, Ministry of Agriculture, Environment and Plant Protection Institute, Chinese Academy of Tropical Agricultural Sciences, Haikou, China

**Keywords:** *Megalurothrips usitatus* (Bagrall), *Beauveria brongniartii*, *Akanthomyces attenuatus*, transcriptome, immune-related genes

## Abstract

*Megalurothrips usitatus* (Bagrall) is an important pest of legumes worldwide, causing great economic loss every year. *Beauveria brongniartii* and *Akanthomyces attenuatus* have shown considerable pathogenicity against *M. usitatus* in our previous studies. The medial lethal concentration (LC_50_) and the sublethal lethal concentration (LC_25_) of *B. brongniartii* isolate SB010 against *M. usitatus* were 8.38 × 10^5^ and 1.73 × 10^5^ conidia mL^−1^, respectively, whereas those of *A. attenuatus* isolate SCAUDCL-53 against *M. usitatus* were 4.37 × 10^5^ and 2.97 × 10^4^ conidia mL^−1^, respectively. This study reports the transcriptome-based explanation of the stress responses of *M. usitatus* following the application of *B*. *brongniartii* and *A. attenuatus*. The analysis of the transcriptomic data revealed the expression of 254, 207, 195, and 234 immunity-related unigenes by *M. usitatus* in response to *B*. *brongniartii* LC_50_ (SB1), *B*. *brongniartii* LC_25_ (SB2), *A. attenuatus* LC_50_ (V1), and *A. attenuatus* LC_25_ (V2), respectively. The biological function and metabolic pathway analyses showed that these unigenes were mainly related to pattern recognition receptors, information transduction factors, and reaction factors, such as scavenger receptor, cytochrome b5, cuticle protein, lysozyme, and serine protease.

## Introduction

*Megalurothrips usitatus* (Bagnall), belonging to the suborder Thysanoptera, is a pest that is a major threat to legumes (Du et al., 2019; Wang et al., 2019; Seetharamu et al., 2020). The flowering period of crops is particularly vulnerable to damage by *M. usitatus*, which causes huge economic losses to crops due to the deformity of and scars on petals and fruits (Zhang et al., 2007; Tang et al., 2015). Chemical control has long been the most preferred method of pest control; however, thrips are particularly difficult to control with insecticides because they hide in small spaces on plants and quickly develop resistance to insecticides. Furthermore, the excessive use of pesticides results in the development of pesticide residues in cowpea, which seriously harms the development of the cowpea industry (Gao et al., 2012, 2018; Fang et al., 2016; Liu et al., 2020). Therefore, it is necessary to find more environmentally friendly measures for the management of *M. usitatus* (Huang et al., 2020). Entomopathogenic fungi can cause diseases in insects. At present, there are about 90 genera and more than 750 species of fungi known to cause disease, which are classified as Chytridiomycota, Ascomycota, Basidiomycota, and the subphylum Entomophthoromycotina. These mainly include *Beauveria bassiana* (Balsamo) Vuillemin, *Metarhizium anisopliae* (Metschnikoff), *Isaria fumosoroseus* (Wize), *Lecanicillium lecanii* (Zimmerman), *Aschersonia* spp., Entomophthorales spp., *Nomuraea rileyi* (Farlow) Samson, etc. (Fan, 2011; Lei et al., 2016; Ma et al., 2016; Rajula et al., 2020). *Beauveria* spp. are the most widely used biological control agent against many pests, and *Akanthomyces attenuatus* has been extensively studied in the control of pests, such as mosquitoes, aphids, whitefly, and thrips (Montalva et al., 2017; Yu et al., 2019; Amobonye et al., 2020; Woo et al., 2020). Yang et al. (2020) found that conidial suspensions, as well as crude protein extracts of *Beauveria brongniartii* isolate SB010 and *A. attenuatus* isolate SCAUDCL-53, were pathogenic against *M. usitatus*.

When defending against external influences, insects depend exclusively on their strong and complex innate immunity (i.e., cellular and humoral immunity) because they lack acquired immunity (Kleino and Silverman, 2014). For example, insect body fat, cuticle protein, and serine protease play a role in the insect response to pathogenic fungi (Chen et al., 2012; Lee et al., 2018). However, the mechanisms through which *M. usitatus* regulates its immune response when defending against pathogenic fungi are currently unclear, and no previous studies, to the best of our knowledge, have been conducted on its immune system.

To better control *M. usitatus* using *B. brongniartii* isolate SB010 and *A. attenuatus* isolate SCAUDCL-53, understanding the immune responses of *M. usitatus* against these two fungi is necessary. Therefore, in this study, *M. usitatus* was exposed to the sublethal lethal concentration (LC_25_) and medial lethal concentration (LC_50_) of SB010 and SCAUDCL-53, respectively, followed by the RNA sequencing analysis to explore the immune response of *M. usitatus* to the application of *B. brongniartii* and *A. attenuatus*.

## Materials and Methods

### Insect Cultures

Adults of *M. usitatus* were collected from a cowpea field at the South China Agricultural University, Guangzhou, China. The collected insects were subsequently reared under laboratory conditions by the bean pod rearing method (Espinosa et al., 2005). The insect colonies were kept at 26 ± 6°C, 70 ± 5% relative humidity (RH), and 16:8 h (light/dark) photoperiod in a climate control chamber. Newly emerged adult females were used for the fungal bioassay studies.

### Fungus Culture and Preparation of Conidial Suspension

The tested strains (i.e., SB010 and SCAUDCL-53) were obtained from the Engineering Research Center of Biological Control, South China Agricultural University, China.

The strains were cultured on the potato dextrose agar plates for 7 days under laboratory conditions. A sterile cell scraper was used to scrape the surface of the mycelium into sterile deionized water with 0.05% Tween 80 to obtain the conidia, which were counted with a hemocytometer (Qian Yihua Glass Instruments Co. Ltd., China.) under a compound microscope (Ningbo Shunning Instruments Co. Ltd., China) at 40 × magnification to calibrate a conidial suspension of 1 × 10^8^ conidia mL^−1^. The lower conidial concentrations (1 × 10^7^, 1 × 10^6^, 1 × 10^5^, and 1 × 10^4^ conidia mL^−1^) for the bioassay studies were prepared by serial dilution.

### Bioassay Method

Centrifuge tube residual bioassays were carried out to evaluate the toxicity of *B. brongniartii* isolate SB010 and *A. attenuatus* isolate SCAUDCL-53 against the *M. usitatus* adults under laboratory conditions (Yang et al., 2020). Both the bean pods (1 cm length) and centrifuge tubes (9 mL) were individually immersed in conidial suspensions of different concentrations (1 × 10^8^, 1 × 10^7^, 1 × 10^6^, 1 × 10^5^, and 1 × 10^4^ conidia mL^−1^) for 2 h. Bean pods and centrifuge tubes treated with sterile ddH_2_O containing Tween 80 served as a control. After drying under sterilized conditions, the bean pods were placed in centrifuge tubes, and *M. usitatus* adults (i.e., 20 individuals) were released into the centrifuge tubes. Each tube was sealed with a cotton plug and kept at 26 ± 6°C, 70 ± 5% R.H., and 16:8 h (L:D) photoperiod in a climate control chamber. Each treatment was set up with four replicates. The mortality data were percent-transformed and subjected to the probit analysis to calculate the LC_50_ and LC_25_ of the two strains. The LC_25_ and LC_50_ of *B*. *brongniartii* and *A. attenuatus* were prepared for later experiments.

### Treatment With *B. brongniartii* and *A. attenuates*

Bean pods (1 cm length) and centrifuge tubes (9 mL) were immersed in the LC_50_ and LC_25_ of *B. brongniartii* and *A. attenuatus* for 2 h, respectively, followed by drying under sterilized conditions. Bean pods and centrifuge tubes treated with sterile ddH_2_O containing Tween 80 served as a control. After drying, bean pods were placed in centrifuge tubes, and *M. usitatus* adults (i.e., 100 individuals) were released into the centrifuge tubes. Each tube was sealed with a cotton plug and kept at 26 ± 6°C, 70 ± 5% R.H., and 16:8 h (L:D) photoperiod in a climate control chamber. Each treatment was set up with four replicates. The insects treated with the LC_50_ and LC_25_ of *B. brongniartii* were named as SB1 and SB2, while the insects treated with the LC_50_ and LC_25_ of *A. attenuatus* were named as V1 and V2, respectively. The control treatment was named as CK.

### Collecting of Tested Insects and RNA Extraction

After 3 days of treatment, the surviving adults were collected in 1.5-mL Eppendorf (EP) tubes, frozen in liquid nitrogen, and stored at −80°C. The total RNA was extracted according to the instructions of the manufacturer of the TRIzol Reagent (Invitrogen, Japan). The quality of the extracted RNA was measured by 1% agar gel electrophoresis and NanoDrop One UV spectrophotometer (Thermo Fisher Scientific, MA, USA).

### Construction and Sequencing of the cDNA Library

After total RNA extraction, mRNAs were enriched with Oligo(dT)-containing magnetic beads treated with DNase I followed by the addition of the fragmentation buffer to break the mRNA into short pieces, which were used as templates with six-base random primers (Random Hexamers) to synthesize the first cDNA chain. The RNA enzyme and DNA polymerase I were added to synthesize the second chain of cDNA. After kit recycling, sticky end repair, addition of 3′ end with A tail, and connection of sequencing joint, the sizes of the desired fragments were selected, and the cDNA library was finally obtained by the PCR enrichment. The Illumina sequencing platform (USA) was used to sequence the library after it was qualified by the Agilent 2100 Bioanalyzer (Agilent Technologies, USA) and ABI StepOnePlus Real-Time PCR System (Bio-Rad, USA).

### Assembly and Annotation of the Transcriptome

Raw reads were filtered and assembled through Trinity (De Novo assembly software). The assembled sequences were removed from redundancy and spliced through TGICL to obtain the longest non-redundant unigene set. Further statistical analysis and quality control were performed on the unigene set. The unigene set obtained by De Novo and clustering was compared with the database by BLAST for functional annotation. The annotation database included nucleotide sequence (NT), non-redundant protein sequences (NR), clusters of orthologous groups (COG), kyoto encyclopedia of genes and genomes (KEGG), and SwissProt, and the unigene set was annotated by Blast2GO based on the annotation results of NR. To understand the transcriptome database of the response of *M. usitatus* to entomopathogenic fungus, 15 digital gene expression (DEG) marker libraries were constructed, among which the transcription information of *M. usitatus* were treated with two different pathogenic fungi and the blank control group.

### Digital Gene Expression Profile Analysis

According to the constructed cDNA library data, the DEG map was used to identify differentially expressed genes. We regarded the genes with a false discovery rate ≤ 0.001 and |fold change| ≥ 2 as differentially expressed genes. Genes with similar patterns of expression often have similar functions. The R toolkit pheatmap software (USA) was used for the cluster analysis of differentially expressed genes and experimental conditions. The Gene Ontology (GO) sequencing was used for the GO enrichment analysis. The main difference between this method and the ordinary hypergeometric distribution is that the influence of gene length preference can be eliminated so that the GO term of true enrichment can be calculated more accurately. By combining the above analysis with the KEGG pathway, GO enrichment analysis, and pattern clustering of DEGs, the GO functional categories and the pathways with significant enrichment of differentially expressed genes were obtained for further analysis.

### Quantitative Reverse Transcription PCR Verification on DEGs

Primers for differentially expressed genes and the reference gene were designed by using Primer Premier 5 software (Premier, Canada). In total, 32 DEGs were selected for quantitative reverse transcription PCR (qRT-PCR) to validate the DEGs. SYBR Premix ExTaq (Perfect Real Time, Takara Bio Inc., Japan) was used for qRT-PCR for the final reaction. The total volume of the qRT-PCR system was 20 μl, including 0.4 μl of the primer volume (i.e., forward/reverse), 1 μl of 10-fold cDNA, 8.2 μl of nuclease-free water, and 10 μl of 2 × Perfect Start TM Green qPCR SuperMix. qRT-PCR was performed on a Bio-Rad (CFX96, USA) iQ5 thermocycler using the temperature conditions of initial denaturation at 94°C for 30 s, followed by 39 cycles of 94°C for 5 s, 55°C for 30 s, and 72°C for 10 s, and then the melt curve at 65°C to 95°C with an increment of 0.5°C for 0.05 s. α-tubulin (TUBA) was used as the reference gene, and their relative expression was calculated using the 2^−ΔΔCq^ method (Guo et al., 2019).

## Results

### Virulence of *B. brongniartii* and *A. attenuatus* Against *M. usitatus*

The LC_25_ and LC_50_ of *B. brongniartii* isolate SB010 after 5 days were 1.73 × 10^5^ and 8.38 × 10^5^ conidia mL^−1^, respectively (Table 1). The LC_25_ and LC_50_ of *A. attenuatus* isolate SCAUDCL-53 after 5 days were 2.97 × 10^4^ and 4.37 × 10^5^ conidia mL^−1^, respectively (Table 1).

**Table 1:** The LC_25_ and LC_50_ values for *B. brongniartii* isolate SB010 and *A. attenuatus* isolate SCAUDCL-53 against *M. usitatus* after 5 days of application.

**Strains**	**LC_50_ (conidia mL^−1^) (95% confidence limit)**	**LC_25_ (conidia mL^−1^) (95% confidence limit)**
SB010	8.38 × 10^5^ (6.23 × 10^4^-7.53 × 10^7^)	1.73 × 10^5^ (26.36-1.27 × 10^6^)
SCAUDCL-53	4.37 × 10^5^ (5.77 × 10^4^-2.42 × 10^6^)	2.97 × 10^4^ (4.48 × 10^2^-1.66 × 10^5^)

### Assembly and Sequencing of the Transcriptome

A total of 45.8–59.7 million raw reads were obtained from 15 samples. Before mapping, the adapters and low-quality reads were filtered to produce 44.8–57.9 million clean reads per sample. Clean reads were assembled by the assembly software. Then, contigs were obtained first, followed by transcripts, and finally, 70,929 unigenes were obtained with an average length of 1,279.38 bp. Among them, 24,168 (34.1%) unigenes were longer than 1,000 bp, and the length of N50 was 2,829 bp. According to the length distribution of *M. usitatus* unigenes, the largest gene length was 200–600 bp, the second largest gene length was 600–1,000 bp, and the smallest gene length was 1,400–2,000 bp (Table 2).

**Table 2:** cDNA library assembly for *M. usitatus*.

**Length range**	**Unigenes**
200–600	37,027 (52.20%)
600–1,000	9,734 (13.72%)
1,000–1,400	5,275 (7.44%)
1,400–2,000	5,051 (7.12%)
2,000+	13,842 (19.52%)
Total_Number	70,929
Max_Length	27,319
Min_Length	200
Mean_Length	1,279.38
N50_Length	2,829
N90_Length	458

### Functional Annotation of the Unigenes

**Figure 1:**
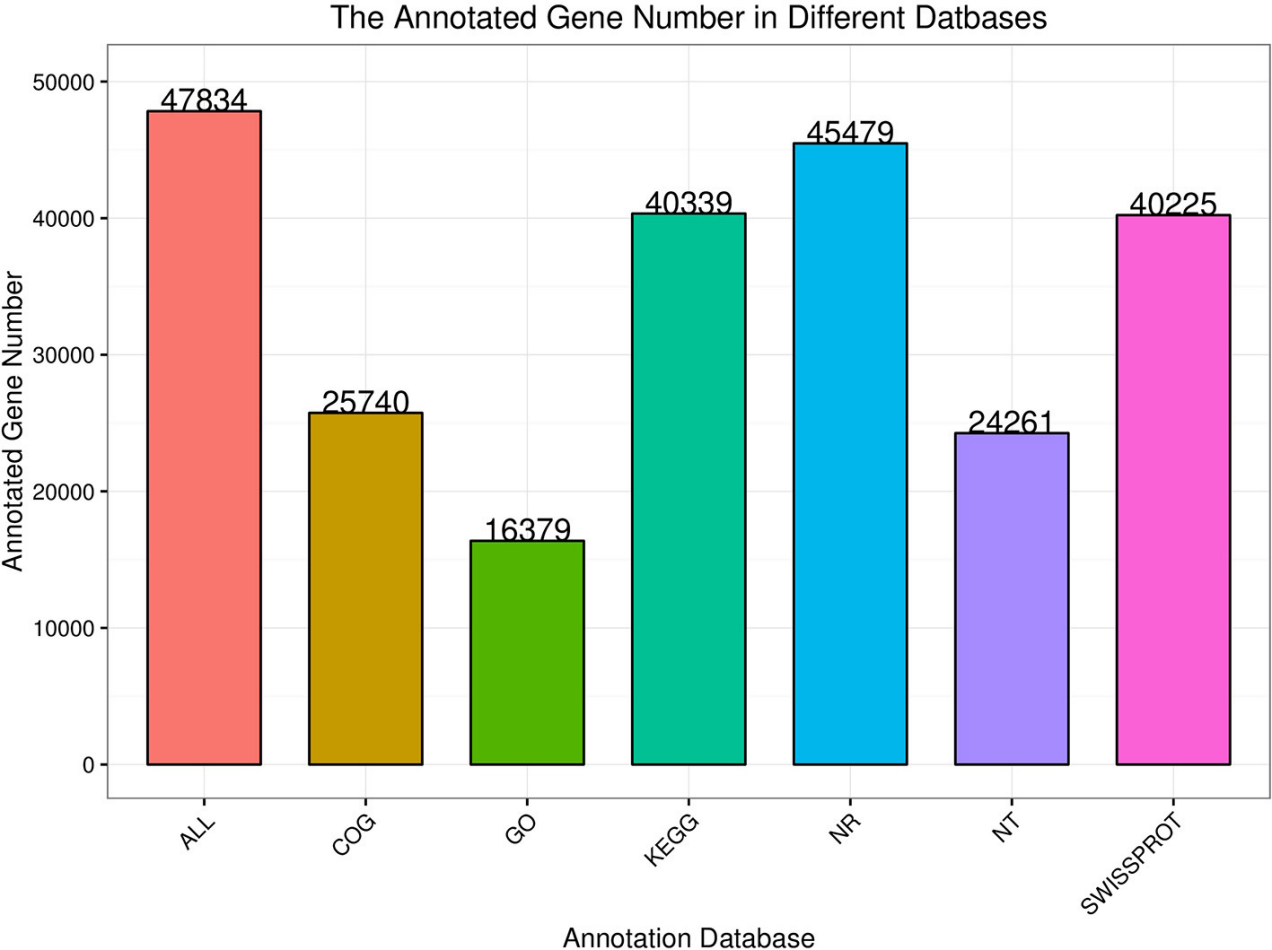
Gene annotations of each database.

There were 47,835 unigenes annotated functionally, of which 25,740 were annotated in the COG database, 16,379 were annotated in the GO database, 40,339 were annotated in the KEGG database, 45,479 were annotated in the NR database, 24,261 were annotated in the NT database, and 40,225 were annotated in the SwissProt database (Figure 1).

**Figure 2:**
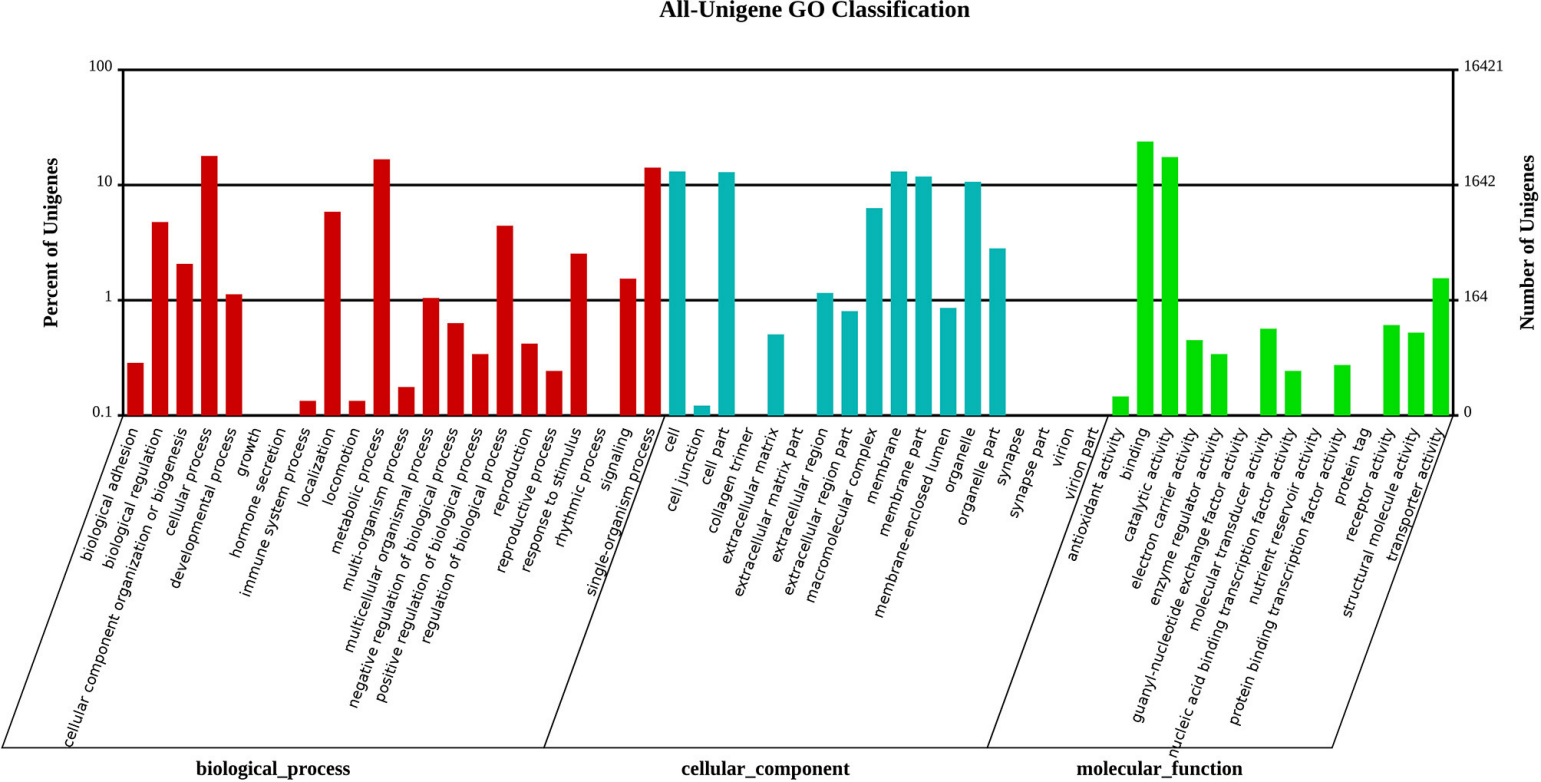
Functional annotation of the assembled sequences based on the Gene Ontology (GO) categorization.

The Gene Ontology analysis was used to predict the function of the protein of *M. usitatus*. Generally, the GO terminology was divided into three categories (Figure 2), namely, biological processes, molecular functions, and cellular components. The number of unigenes related to “biological processes” and “cellular components” was similar, with 12,260 and 12,214, respectively (i.e., 38.25 and 38.11% of the total), whereas the “molecular function” was the least observed (i.e., 7,579, 23.46%). The COG classification was also used to analyze the presumed protein function, and 25,740 unigenes were divided into 25 COG classes according to their function (Supplementary Figure 1).

There were 40,339 unigenes distributed over 337 annotated pathways in the KEGG database. The numbers of unigenes involved in different pathways were varied. Those pathways with <1,000 unigenes were classified into one category (others), and the remaining 10,734 unigenes were classified into 19 categories. The metabolic pathway (ko01100) was the largest category with 6,624 unigenes. Few unigenes were involved in “RNA processing and modification,” “Cell motility,” and “Defense mechanisms” (Supplementary Figure 2).

### Identification of Immune-Related Genes

In the groups, such as CK-VS-SB1, CK-VS-SB2, CK-VS-V1, and CK-VS-V2, 254, 207, 195, and 234 immune-related genes were obtained, respectively. According to the results, more differentially expressed immune-related genes were observed for a higher concentration of *B*. *brongniartii* (SB1). However, the opposite result was obtained for *A. attenuatus*.

### Analysis of Expression Profile

The fragments per kilo bases per million reads (FPKM) values for the unigenes were obtained, and the Log_2_ fold change (FC) or Log_2_Ratio (i.e., FPKM of the experimental group/FPKM of the control group) was calculated later. We found that in the groups such as CK-VS-SB1, CK-VS-SB2, CK-VS V1, and CK-VS-V2, there were 3,231, 2,698, 2,119, and 2,893 differentially expressed genes, respectively. The cluster analysis of differentially expressed genes showed that the Log_2_FC values of most genes in the treatment group were negative, which indicated that these genes were downregulated compared with those in the control group (Supplementary Figure 3).

**Figure 3:**
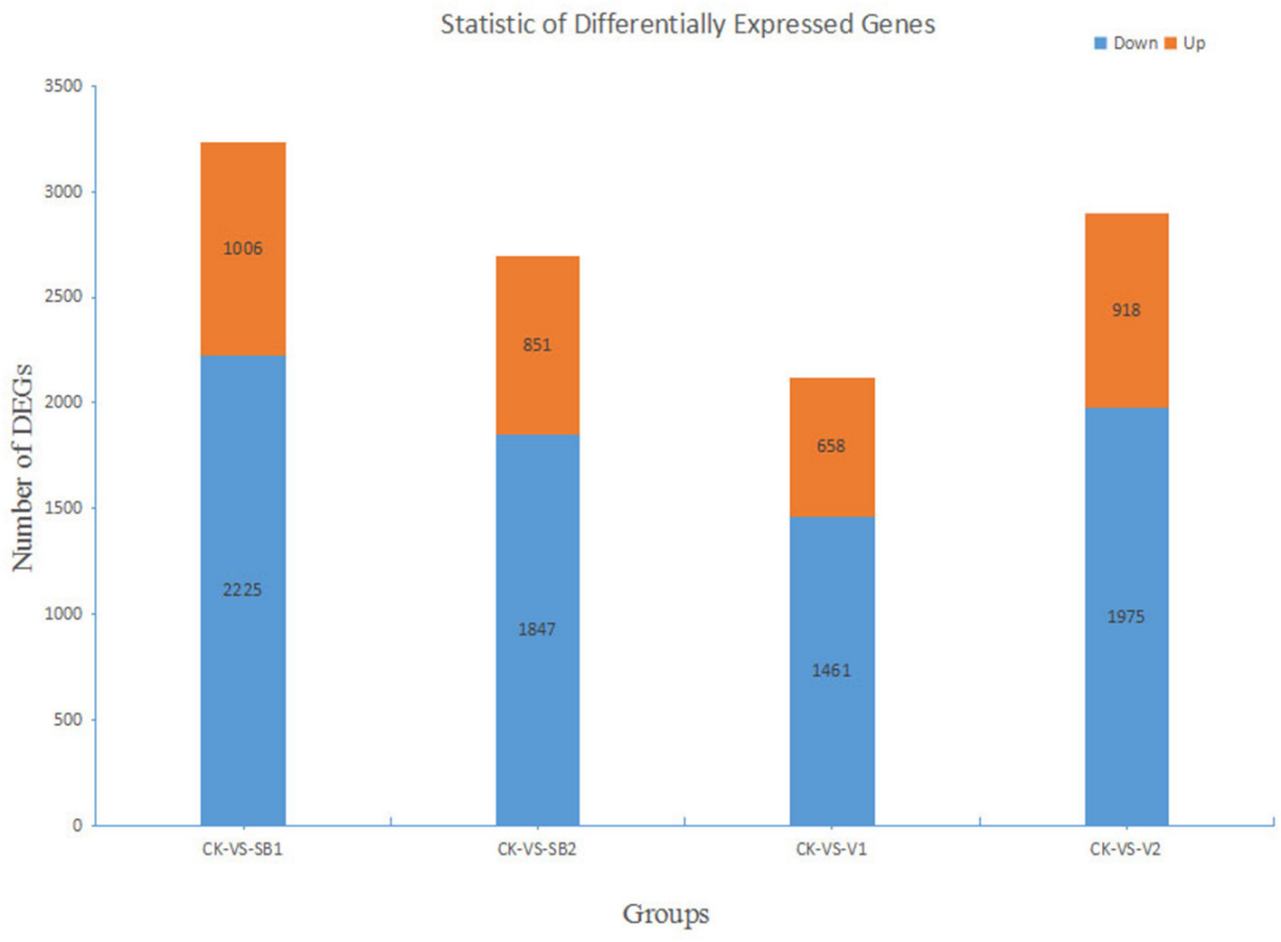
Statistical analysis of digital gene expressions in different groups.

The results showed that there were 2,225 downregulated genes and 1,006 upregulated genes in the CK-VS-SB1 group, among which the genes with the Log_2_Ratio values in the range of −5 to −1 were in the majority (i.e., 2,184, 67.60%), and the number of genes with a Log_2_Ratio value in the range of 2–9 was 562 (17.39%). There were 1,847 downregulated genes and 851 upregulated genes in the group CK-VS-SB2, among which, the genes with Log_2_Ratio values in the range of −5 to −1 were in the majority (1,847, 68.46%), and the numbers of genes with Log_2_Ratio value in the range of 2–9 are 272 (10.08%). There were 1,461 downregulated genes and 658 upregulated genes in the CK-VS-V1 group, among which, the genes with Log_2_Ratio values in the range of −5 to −1 were in the majority (1,419, 66.97%), and the number of genes with a Log_2_Ratio value in the range of 2–9 was 287 (13.54%). There were 1,975 downregulated genes and 918 upregulated genes in the CK-VS-V2 group, among which, the genes with Log_2_Ratio values in the range of −5 to −1 were in the majority (1,930, 66.71%), and the number of genes with a Log_2_Ratio value in the range of 2–9 was 354 (12.24%) (Figure 3). In the four groups, the unigene IDs of the most upregulated genes were CL8532.Contig1_All (by 7.68-fold), CL1603.Contig8_All (by 8.88-fold), CL1726.Contig1_All (by 7.64-fold), and CL1603.Contig8_All (by 8.32-fold), respectively (Supplementary Table 1). The unigene IDs of the most downregulated genes were CL8474.Contig2_All (by −8.87-fold), CL366.Contig6_All (by −7.87-fold), CL1664.Contig3_All (by −9.8-fold), and CL366.Contig6_All (by −7.88-fold), respectively (Supplementary Table 2).

We analyzed some genes that may participate in the response to *B*. *brongniartii* (i.e., SB1 and SB2) and *A. attenuatus* (i.e., V1 and V2) infections (Table 3). Among SB1, the function annotation identified that the cytochrome b5 (CYT-B5) was the highest upregulated gene with a Log_2_Ratio value of 7.68, followed by the hypothetical protein. Among SB2, the function annotation identified that the CYT-B5 was the highest upregulated gene with a Log_2_Ratio value of 7.38, followed by the scavenger receptor. Among V1, the function annotation identified that the CYT-B5 was the highest upregulated gene with a Log_2_Ratio value of 6.82, followed by the hypothetical protein. Among V2, the function annotation identified that the CYT-B5 was the highest upregulated gene with a Log_2_Ratio value of 7.7, followed by the conserved hypothetical protein. This implies that the two genes may directly or indirectly play roles in the response of *M. usitatus* against the infection by *B. brongniartii* and *A. attenuatus*. The differentially expressed genes were mainly involved in several pathways, such as the platelet activation (ko04611), the janus kinase/signal transducers and activators of transcription (Jak/STAT) signaling pathway (ko04630), and the mitogen-activated protein kinase (MAPK) signaling pathway (ko04010).

**Table 3:** Differently expressed unigenes likely related to the immune response of *M. usitatus* after treatment with *B*. *brongniartii* and *A. attenuatus*.

**Groups**	**Genes ID**	**Log_**2**_Fold**	***p*** **-value**	**Nr-annotation**
CK-VS-SB1	CL8532.Contig1_All	7.682691374	7.44E-170	Cytochrome b5 isoform X1
	Unigene28189_All	7.232605504	1.07E-16	Hypothetical protein, partial
	CL1563.Contig1_All	7.195736769	3.23E-17	Scavenger receptor class B
	CL1103.Contig4_All	6.89029466	6.48E-16	Heat shock protein 90-2
	CL812.Contig1_All	6.597832802	1.24E-12	Heat shock 70 kDa protein 4 isoform X2
	CL1547.Contig3_All	6.120244674	1.33E-11	Hypothetical protein TSAR_005269
	CL932.Contig1_All	6.307385796	6.25E-12	Hypothetical protein
	CL4847.Contig1_All	6.407005935	9.43E-13	Hypothetical protein
	Unigene3861_All	2.16137876	1.34E-14	Serine protease inhibitor 42Dd-like isoform X1
CK-VS-SB2	CL8532.Contig1_All	7.380996965	3.07E-157	Cytochrome b5 isoform
	CL1563.Contig1_All	6.959879909	3.88E-18	Scavenger receptor class B member 1-like isoform X1
CK-VS-V1	CL1563.Contig1_All	5.478985966	1.07E-11	Scavenger receptor class B
	CL8532.Contig1_All	6.817440463	1.70E-76	Cytochrome b5 isoform X1
	Unigene31739_All	6.04718163	7.43E-15	Hypothetical protein, partial
	CL6023.Contig3_All	4.005334327	1.94E-11	Hypothetical protein AMK59_2033
	CL1569.Contig14_All	5.109423527	5.47E-10	Heat shock protein cognate 70-1
	CL1523.Contig4_All	2.066643114	1.28E-16	Vitellogenin
CK-VS-V2	CL8532.Contig1_All	7.704830185	2.66E-174	Cytochrome b5 isoform X1
	CL1563.Contig1_All	5.68723684	9.46E-12	Scavenger receptor class B member 1-like isoform X1
	CL2688.Contig3_All	4.664464118	5.02E-10	Hypothetical protein DAPPUDRAFT_307202

### Quantitative Reverse Transcription PCR Validation Study

**Figure 4:**
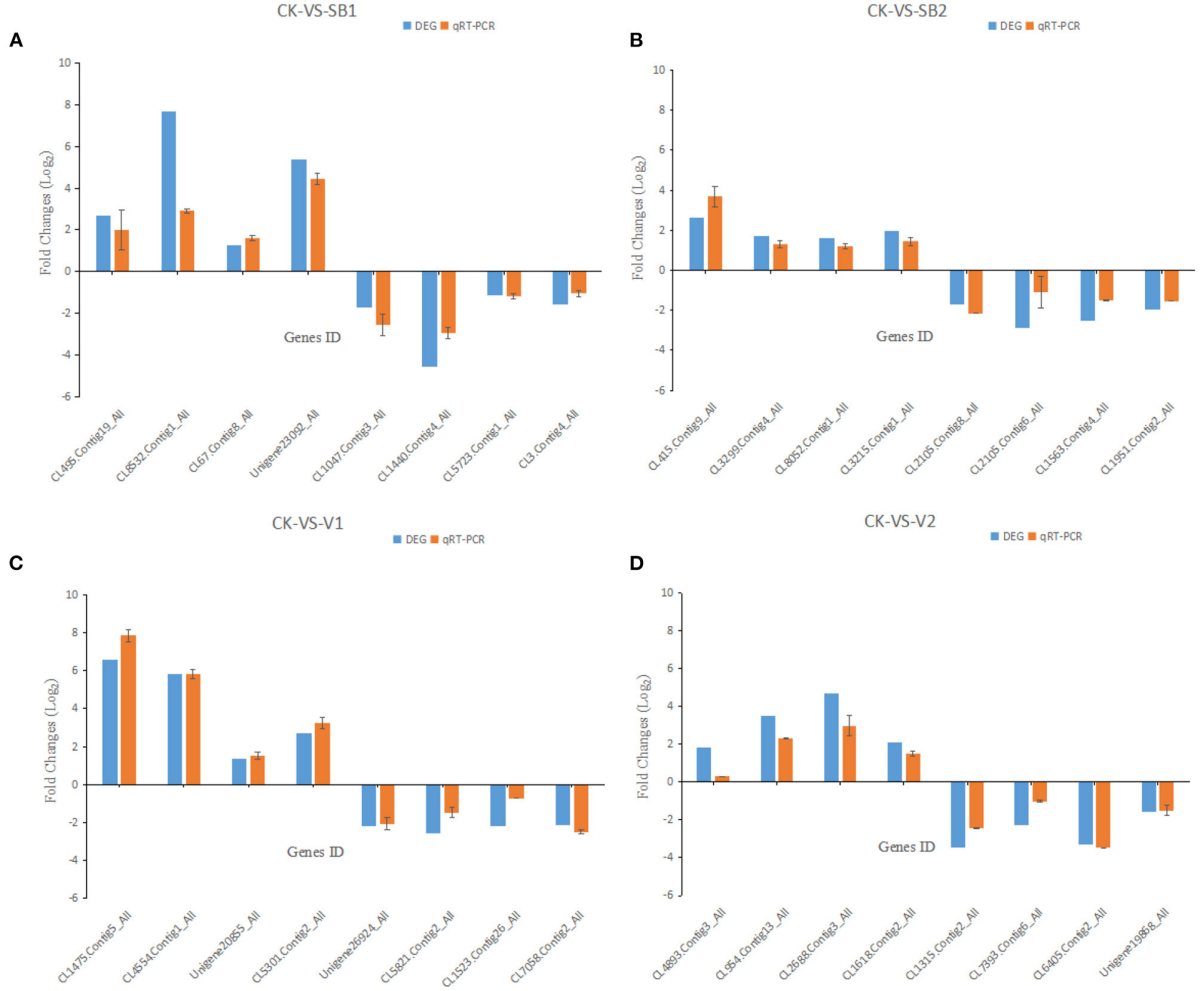
The qRT-PCR results of the expression of genes in *M. usitatus*. **(A)** Treatment with LC_50_ of *B*. *brongniartii*; **(B)** Treatment with LC_25_ of *B*. *brongniartii*; **(C)** Treatment with LC_50_ of *A. attenuates*; **(D)** Treatment with LC_25_ of *A. attenuates*.

The accuracy of DEGs was verified by the qRT-PCR. Selecting 32 genes randomly, they all showed similar expression patterns to the DEG analysis. Therefore, the DEG method is reliable (Figure 4).

## Discussion

In this study, we collected the samples that were treated with the LC_25_ and LC_50_ concentrations of SB010 and SCAUDCL-53 for sequencing and obtaining the transcriptional database. The transcriptional database consisted of 70,929 unigenes. The analysis of the transcriptomic data showed 3,231, 2,698, 2,119, and 2,893 differently expressed unigenes for SB1, SB2, V1, and V2, respectively. There were 254, 207, 195, and 234 immunity-related unigenes for SB1, SB2, V1, and V2, respectively. These genes were related to recognition, signaling transduction, effectors, modulators, and so on. Yang et al. (2020) found that *B. brongniartii* had a good effect on *M. usitatus*, but its intensity and speed were significantly different from those of *A. attenuates*, which had stronger virulence and faster speed. In this study, when the LC_25_ and LC_50_ of the two pathogens were used to treat *M. usitatus*, more immune-related genes were obtained for SB1 than for SB2, while the opposite result was obtained in the treatment of *A. attenuates*. This is probably because a part of the defense system of the host had been damaged, which led to the expression of the immune-related genes beginning to be suppressed when treated with the LC_50_ of *A. attenuates*. In contrast, *B. brongniartii* had a stronger immune system with a higher concentration due to the gentle mode of action. The results of this study are similar to those suggested by Chen (2016).

Cytochrome b5 is an important member of the p450 protein family, which can promote the metabolism of related catabolic enzymes, but its function is currently under debate. Studies on CYT-B5 are mainly focused on mammals and rarely on insects (Feyereisen, 2012; Zheng et al., 2019; Jiao et al., 2020). CYT-B5 is associated with insect resistance but is rarely reported in insect immunity (Scott and Georghiou, 1986; Yu and Nguyen, 1992). In this study of the four treatments, CYT-B5 (unigenes ID: CL8532.Contig1_All) was involved in the platelet activation, and its expression level increased up to 6–7 times, indicating that CYT-B5 had an effect on the response of *M. usitatus* to *B*. *brongniartii* and *A. attenuatus*. The results provide evidence for the discovery of other functions of CYT-B5 in insects.

Scavenger receptors have a wide range of biological functions, such as cellular immune recognition and phagocytosis (Keehnen et al., 2017; PrabhuDas et al., 2017). In this study, many scavenger receptor genes were found, among which, scavenger receptor Type B (unigenes ID: CL1563.Contig1_All) was found in all four treatments with 5- to 7-fold increased expression. This indicated that the recognition plays a significant role in the immune response of *M. usitatus* to pathogen infection. Fujita et al. (2021) found that SR-B1 plays an important role in the endocytosis of HEK293T and the formation of lipid droplets. Zhang (2016) showed that the scavenger receptor expression was upregulated in *Locusta migratoria* after infection by *M. anisopliae*. While studying an increase in the interleukin-4 (IL-4) level in the phagocytosis of necrotic cells by macrophages through scavenger receptor CD36, Chen et al. (2021) found that IL-4-enhanced phagocytosis was mediated by the upregulation of scavenger receptor CD36. Krieger (1997) showed that scavenger receptors can combine with various pathogens and have great effects on host innate immunity and defense. The results of this study are consistent with those in this study, which indicate that scavenger receptors also make a difference to *M. usitatus* immunity.

The cuticle of insects is the first barrier against pathogen infection and plays an indispensable role in maintaining the shape and mobility of the insects (Yu et al., 2016). The cuticle is an envelope that can enclose the insect larva completely and can protect it from the external environment (Delon and Payre, 2004). Chitin, lipids, catecholamines, minerals, and cuticle proteins are the main components of cuticles (Zhu et al., 2016; Shang et al., 2020). Cuticle proteins contribute considerably to insect resistance, drug resistance, and immunity and are induced to strengthen or stabilize the structure of the cuticle to resist the adverse conditions and maintain the survival of insects when they are in challenging conditions (Silva et al., 2012b; Yu et al., 2016). Thickening of the cuticle is one of the mechanisms of insecticide resistance (Silva et al., 2012a). Studies on environmental stress induced the expression of the cuticle protein of *Leptinotarsa decemlineata* and found that potato beetles may increase the deposition of cuticle components at the adult stage to respond to the environmental stress, which enables insects to adapt to new or changing environments (Zhang et al., 2008). Silva et al. (2012b) found that the cuticle protein genes of aphids are upregulated when resisting pesticides. In the four treatments, the expression of cuticle proteins was upregulated, which indicated that the cuticle proteins regulate the immune process of *M. usitatus* through negative regulation. These results are contrary to those of the present study, which may be because different species lead to different regulatory processes of cuticle protein genes. The results of this study provide evidence for the importance of the study of the cuticle protein genes in the immune defense of *M. usitatus*.

Vitellogenin is another important insect protein, which is considered to provide nutrients for the development of yolk (Antúnez et al., 2013; Kodrík et al., 2019; Yang, 2020). However, many studies have shown that vitellogenins play important roles in the immune systems of amphioxus, potato psyllid, honeybees, *Aedes aegypti*, and so on (Raikhel et al., 2002; Antúnez et al., 2013; Shen et al., 2019). In this study, it was also found that the expression of some vitellogenins was upregulated after pathogen treatment, which indicated that vitellogenins are involved in the process of resistance to pathogens in *M. usitatus*. The result provides further evidence that vitellogenin has immunologic functions.

Heat shock proteins (HSPs), which are the intracellular molecular chaperones of naive proteins, are induced under stressful conditions, such as heat, cold, radiation, insecticides, oxidative stress, heavy metal, viruses, bacteria, and fungi, and are known for thermal induction (Shomali et al., 2021). Wojda and Jakubowicz (2007) found that HSP90 may have an effect on the converging pathways involved in the insect immune response and heat shock. Richards et al. (2017) found that HSPs can increase the ability of insects to defend against infection by *B. bassiana* and *Metarhizium brunneum*, improving survival. de Morais Guedes et al. (2005) found that HSP70 and HSP82 were upregulated in the hemolymph of *D. melanogaster* after the immune challenge. In this study, the expression of HSPs was upregulated when defending against two pathogens, namely, HSP70 and HSP90, by up to 6.89 times. The results suggest that the expression of HSPs could be induced by *B. brongniartii* and *A. attenuatus* in *M. usitatus*, strengthening the immune system.

After the pathogen breaks through the insect body wall and enters the blood cavity, it causes death by consuming the nutrition of the host, interfering with metabolism, secreting toxin, and destroying the tissue structure. Among the four treatments, the genes related to integral components of the membrane, nucleus, ribosome, metabolic process, transmembrane transport, regulation of gene expression, and serine-type endopeptidase activity were mostly downregulated. This may be because *M. usitatus* has been unable to adapt to the invasion of pathogens, which leads to many functions of the host being degraded and the host entering a state of death at that time.

## Conclusion

The LC_50_ and LC_25_ of SB010 against *M. usitatus* were 8.38 × 10^5^ and 1.73 × 10^5^ conidia mL^−1^, respectively, whereas the LC_50_ and LC_25_ of SCAUDCL-53 against *M. usitatus* were 4.37 × 10^5^ and 2.97 × 10^4^ conidia mL^−1^, respectively.

This study has provided the transcriptomic response of *M. usitatus* to *B*. *brongniartii* and *A. attenuatus*. The results showed that *B*. *brongniartii* and *A. attenuatus* infection mainly changed the expression of genes, such as scavenger receptor, CYT-B5, cuticle protein, lysozyme, and serine protease. We can use RNA interference techniques to verify the role of these genes. This study will improve our understanding of the defense system of *M. usitatus* against *B*. *brongniartii* and *A. attenuatus*.

## Data Availability Statement

The datasets generated for this study can be found in online repositories. The names of the repository and accession number can be found below: National Center for Biotechnology Information (NCBI) BioProject, https://www.ncbi.nlm.nih.gov/bioproject/, PRJNA731850.

## Author Contributions

JW and SA designed the study and revised the manuscript. YC and BY performed the experiments. YC, BY, and ZL analyzed the data. YY, QT, and WC prepared the materials used in the study. YC wrote the manuscript. All authors read and approved the manuscript.

## Conflict of Interest

The authors declare that the research was conducted in the absence of any commercial or financial relationships that could be construed as a potential conflict of interest.

## Publisher's Note

All claims expressed in this article are solely those of the authors and do not necessarily represent those of their affiliated organizations, or those of the publisher, the editors and the reviewers. Any product that may be evaluated in this article, or claim that may be made by its manufacturer, is not guaranteed or endorsed by the publisher.

## Supplementary Material

Supplementary Image 1 COG function classification of all unigenes.

Supplementary Image 2 Clustering analysis of differentially expressed genes in *M. usitatus* after treatment with the LC_25_ and LC_50_ of *B. brongniartii* and *A. attenuatus*.

Supplementary Image 3 Proportion of each category path.

Supplementary Table 1 Ten most upregulated unigenes in *M. usitatus* after treatment with the LC_25_ and LC_50_ of *B. brongniartii* and *A. attenuatus*.

Supplementary Table 2 Ten most downregulated unigenes in *M. usitatus* after treatment with the LC_25_ and LC_50_ of *B. brongniartii* and *A. attenuatus*.
