# Supplementary figures and images for: Immune-Related Genes of Megalurothrips usitatus (Bagrall) Against Beauveria brongniartii and Akanthomyces attenuatus Identified Using RNA Sequencing
Source: Front Physiol. 2021 Aug 11;12:671599. doi: 10.3389/fphys.2021.671599 (PMC8385781; doi:10.3389/fphys.2021.671599)

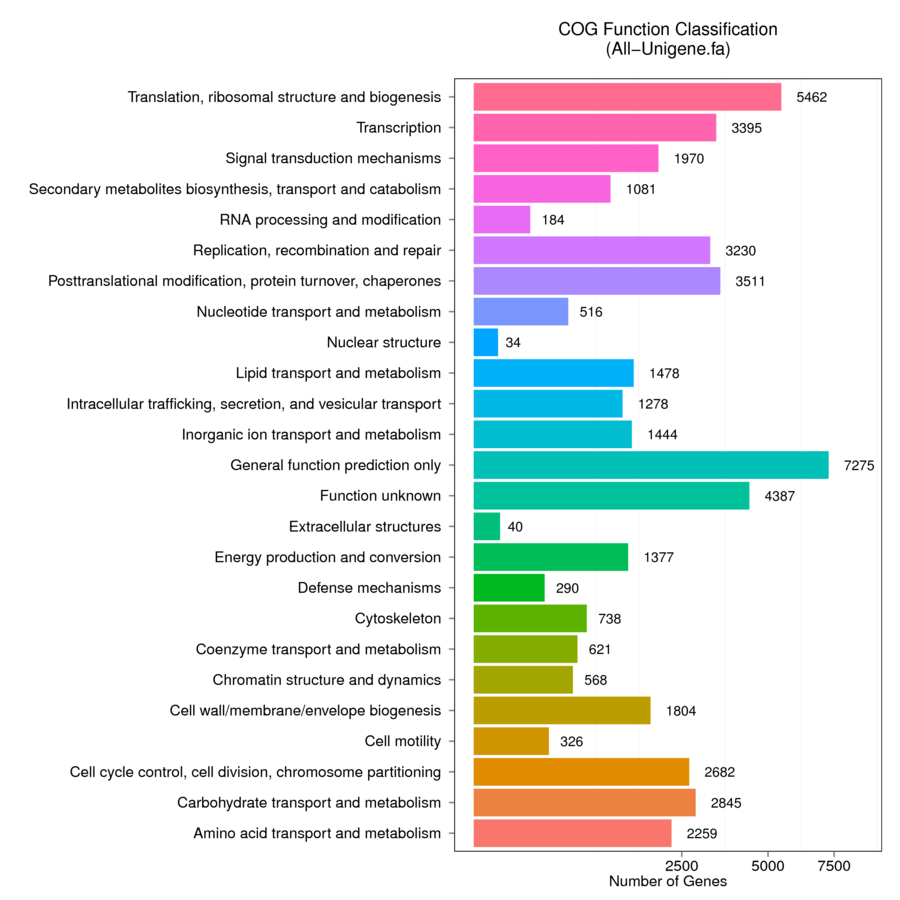

Supplement: Supplementary Image 1 — COG function classification of all unigenes. [file Image_1.PNG]

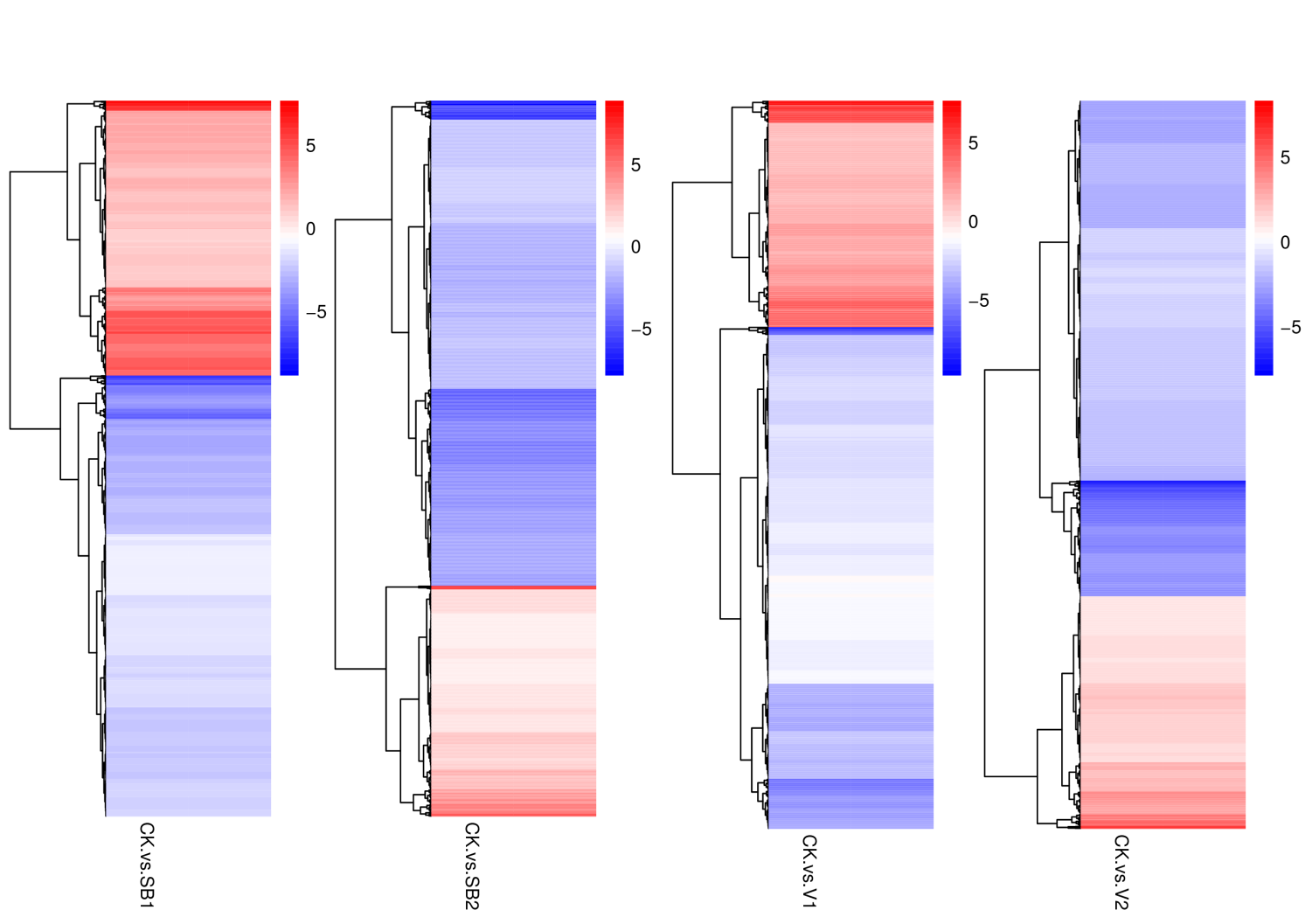

Supplement: Supplementary Image 3 — Proportion of each category path. [file Image_3.png]
