# Supplementary material for: Immune-Related Genes of Megalurothrips usitatus (Bagrall) Against Beauveria brongniartii and Akanthomyces attenuatus Identified Using RNA Sequencing
Source: Front Physiol. 2021 Aug 11;12:671599. doi: 10.3389/fphys.2021.671599 (PMC8385781; doi:10.3389/fphys.2021.671599)
Supplement: Supplementary Image 2 — Clustering analysis of differentially expressed genes in M. usitatus after treatment with the LC25 and LC50 of B. brongniartii and A. attenuatus. [file Image_2.pdf]

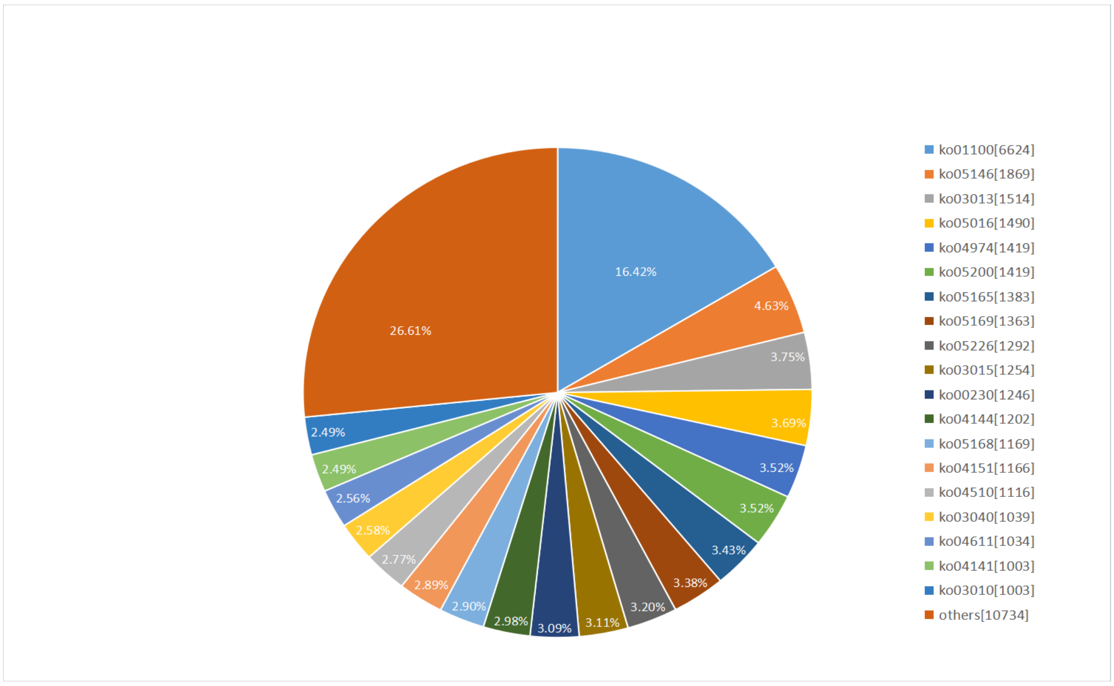

Proportion of each category path

(Notice:ko01100: Metabolic pathways; ko05146: Amoebiasis; ko03013: RNA transport; ko05016: Huntington's disease; ko04974: Protein digestion and absorption; ko05200: Pathways in cancer; ko05165:Human papillomavirus infection; ko05169: Epstein-Barr virus infection;ko05226: Gastric cancer; ko03015: mRNA surveillance pathway; ko00230: Purine metabolism; ko04144: Endocytosis; ko05168: Herpes simplex infection; ko04151: PI3K-Akt signaling pathway; ko04510: Focal adhesion; ko03040: Spliceosome; ko04611: Platelet activation; ko04141: Protein processing in endoplasmic reticulum; ko03010: Ribosome)
